# The Development of Reference Realization and Narrative in an Australian Contact Language, Wumpurrarni English

**DOI:** 10.3389/fpsyg.2016.00043

**Published:** 2016-02-03

**Authors:** Samantha Disbray

**Affiliations:** Cooperative Research Centre for Remote Economic Participation – Remote Education Systems, Northern Institute, Charles Darwin UniversityAlice Springs, NT, Australia

**Keywords:** contact language, Wumpurrarni English, child language acquisition, discourse strategies, reference in narrative, children's development of reference

## Abstract

The development of narrative skill has been investigated extensively in a wide range of languages, cross-linguistically and in multilingual settings (Berman and Slobin, 1994b; Severing and Verhoeven, 2001; Hickmann, 2004; Strömqvist and Verhoeven, 2004). The present study investigates the development of reference realization in narrative among Indigenous children in a remote urban township in Central Australia. The children, aged between 5 and 14 years, are speakers of a contact language, Wumpurrarni English. Language development is rarely investigated among speakers of minority languages, whose language development is often appraised in the majority language, with little attention to language performance in the speaker's home variety. The present study addresses this gap through a fine-grained qualitative analysis of the development of reference in narrative, drawing on a complex stimulus and a model of discourse strategy. The results show (a) a developmental trajectory similar to that found in other languages, with children aged eight and under producing simpler and less globally organized narratives than older speaker groups, and (b) vulnerability to the changing demands of the stimulus among these younger speakers. In addition, a subset of narrations were produced in “school variety,” a style more like Standard Australian English. The results for this set showed that the narrative content and global organization of the productions by 10- and 12-year-olds were more similar to the productions of younger children, than like-aged speakers, who narrated in their home variety. Analysis of speaker responses to two factors of complexity, the stimulus and code choice, illuminated mechanisms for discourse production and development, and suggest that constructing discourse requires co-ordination of an underlying schema and on-line construction of a particular story, through the deployment of linguistic devices in a particular narrative context. The analysis showed that these two skills are tightly interdependent, and indeed co-constructing.

## Introduction

Constructing discourse such as narration is a multidimensional skill with a long developmental path (Hickmann, 2003, 2004; Berman, 2004, 2008; Mäkinen et al., 2014). Language development is shaped by the complex interplay of linguistic features and perceptual, cognitive, and communicative factors (Karmiloff-Smith, 1992). Discourse provides a rich site for investigating this interaction, such that Berman has characterized it as a “meeting ground of developing linguistic knowledge and general cognitive growth” (2008, p. 736). Producing a narrative text requires coordination of a number of knowledge schemas, which children must master. They must formulate and represent narrative content, build a macrostructure (McCabe and Peterson, 1991; Stein and Albro, 1997; Shiro, 2003) via story schema and microstructure via cohesive language (Clancy, 1992; Hickmann et al., 1995; Orsolini et al., 1996; Wong and Johnston, 2004), while taking consideration of the listener's needs, which change as the story progresses (Johnston, 2008).

The development of narrative skill has been investigated extensively in a wide range of languages, cross-linguistically and in multilingual settings (Berman and Slobin, 1994b; Hickmann, 2004; Strömqvist and Verhoeven, 2004). However, few studies have focussed on narrative development in minority languages, on language specific features and on the developmental trajectory of children in their home language or variety. Indeed most often children are appraised in the dominant language, without attention to their home variety (Severing and Verhoeven, 2001; Horton-Ikard, 2009; Mills, 2015). Evaluations of the school dialect are assumed to represent the child's cognitive development, without evidence (Gorman et al., 2011; Burns et al., 2012).

In Australia, there is a growing research base describing the bi- and multi-varietal repertoires of Indigenous children in complex dynamic contact settings (Disbray and Wigglesworth, 2008; Meakins, 2008; Morrison and Disbray, 2008; Simpson and Wigglesworth, 2008; O'shannessy, 2009; Dixon, 2015; Vaughan et al., 2015). Angelo and colleagues have cast light on the “invisibility” of contemporary Indigenous language varieties, particularly in the education sphere, where high stakes testing regimes in Standard Australian English overshadow attention to children's linguistic abilities in their home varieties (Angelo, 2012, 2013; McIntosh et al., 2012; Sellwood and Angelo, 2012; Angelo and Carter, 2015). One set of studies has described culturally specific narrative schema in the personal narratives and recounts by children who speak West Australian Aboriginal English speaking (Malcolm and Sharifian, 2002, 2005; Sharifian, 2010). However, few studies have applied developmental methods and related findings to the research literature on child language development to contact languages (cf. O'shannessy, 2006, 2008, 2011).

The present study addresses this gap by examining the development of reference and narrative among Australian Indigenous children speaking in their Home Variety (HV), Wumpurrarni English. A small set of narrations in children's second language/dialect, Standard Australian English, are also examined. These allow a preliminary exploration of the differences in development in the two varieties and highlight the need for greater attention to children's HV in appraisals of their language and cognitive development.

## Approaches to narrative development

The present study of reference realization and discourse development draws on various theoretical perspectives. First, a *developmentally motivated approach* casts light on the ways that children come to manipulate forms learned early for different functions, in a growing variety of contexts. This requires socio-cognitive development and rich pragmatic, linguistic experience over time, reflected in Karmiloff-Smith's (1992) model of re-representation. This posits that increased cognitive flexibility and generality is achieved as children come to re-represent knowledge in increasingly accessible forms, including linguistic knowledge. Next, a *functional approach* to language acquisition (Karmiloff-Smith, 1981; Hickmann, 2003, 1987) attends to the interrelation between (linguistic) forms and (discourse) functions and this interrelation provides a point of departure to investigate language knowledge, development and use (Slobin, 2001; Hickmann, 2004). A further approach derives from Slobin's exploration of the relation between thought and language as “*thinking for speaking”* (Slobin, 1996, 2004). This posits that the grammar of a language provides a “set of options for schematising experience for the purposes of verbal expression” (1996, p. 75). As children learn a given language, they learn the habitual thought or dispositions and rhetorical styles of the language, which provide available structures and features. The model seeks to explain the mental processes that are involved in planning and constructing linguistic utterances in discourse from this available trove, a particular way of thinking, which is “*thinking for speaking.”*

Studies of bilingual language development exploring the impact of patterns developed in first language acquisition on subsequent language learning and production have also been informed by “*thinking for speaking*” (Benazzo and Andorno, 2010; Dimroth et al., 2010). Pertinent also to the present study, the investigation of narrative development among bi- and multilingual speakers foregrounds socio-cultural factors in multilingual contexts, such as the “complicated sociolinguistic position of minority groups,” and the interplay of variables such as language input, proficiency and speaker's cultural orientation (Verhoeven and Strömqvist, 2001, p. 7). The *socio-cultural context* provides a final approach, important to language development in a dynamic contact setting, such as the site of the present study.

Focussing on the processing task in narrative production and establishing further points of complexity, Stavans (2001, p. 341) posits that the multilingual speaker faces “a reconciliation task between the linguistic worlds available to him/her. Such reconciliation entails ‘juggling’ not only conceptual information but also linguistic and cultural information as well.” According to Stavans, “manipulation of a multi-communication system implies constant and very controlled monitoring of all the cognitive, linguistic and cultural channels. Attending to all these channels of information, in turn implies a certain degree of negotiation where trades and concessions are made” (Stavans, 2001, p. 341). Such observations point to the interplay of linguistic features and perceptual, cognitive and communicative factors discussed above.

## Reference realization in children's narrative development

The specific discourse function investigated in the present study is reference realization. Realizing and managing reference in narration requires knowledge of linguistic forms such as nouns, pronouns, and zero anaphors, and mobilizing these forms for a range of functions; introducing characters and then moving them through the story, maintaining reference where reference persists and reintroducing characters when the referent re-enters the immediate discourse, while allowing listeners to easily follow these referential moves and with them, the unfolding plot.

Characters are generally introduced with full lexical nouns marking newness. In English for instance, the indefinite article “a” achieves this for singular nouns. Other means of identification and newness marking include proper nouns and clause structure, and differences across languages and genres exist (Hickmann, 2003; Jokinen and Wilcock, 2006). Once established, reference can be maintained with minimal linguistic material such as a pronoun, realized in full or unrealized as zero anaphora, avoiding redundancy, flagging the high accessibility of the referent in question and creating locally cohesive links across clauses. Introduced referents may be reintroduced in the discourse, and this switch is marked with lexically full expressions, which avoid ambiguity and create a link to the antecedent reference, creating a link to the previous discourse. Thus, referential phrases serve to disambiguate reference, create local, and discourse-wide links, and these referential moves contribute to global coherence (Berman and Slobin, 1994a; Shapiro and Hudson, 1997; Hickmann, 2004).

Developmental changes have been observed in the ways that children introduce new referents, with a number of studies indicating that marking newness for this discourse function is not systematically mastered until around age 10 (Wigglesworth, 1990, 1997; Clancy, 1992; Kail and Hickmann, 1992; Hickmann and Hendriks, 1999; Bavin, 2000). Differences are observed across this age span in switching between introduced referents or reintroducing referents (Wong and Johnston, 2004), while maintaining reference across adjacent clauses is more uniform across ages (Bamberg, 1987; Hickmann et al., 1995; Orsolini et al., 1996; Hickmann and Hendriks, 1999; Jisa, 2000; Hickmann, 2003). Mäkinen et al. (2014) considered referential cohesion in relation to other elements, such as coherence through story content, in narrations by 4- to 8-year-old Finnish speakers. They found that mastery of cohesion still developing among the eldest group, suggesting, in line with Johnston (2008), that “the precise use of cohesive devices in narration is sophisticated only after the management of story content is established” (Mäkinen et al., 2014, p. 38).

### Discourse strategies and narrative development

Further studies have scrutinized the interactions between the form-function pairings for both reference maintenance and switch, highlighting the multifunctional nature of pronouns and anaphora in marking inter- and intra-sential relations, and in organizing discourse (Karmiloff-Smith, 1985; Bamberg, 1987; Hemphill et al., 1991; Wigglesworth, 1997). In her set of seminal studies, Karmiloff-Smith (1981, 1983, 1985) observed that children aged between 5 and 10 years of age managed reference across a narrative in non-adult but patterned ways. She explored interaction between the ways that children managed reference and narrative content, particularly between the realization of reference for local cohesion through particular linguistic forms according to status of the character (main/secondary), and the ways that this manipulation contributed to discourse organization overall.

Recognising that co-ordinating the various knowledge schemes represents both a cognitive and linguistic task, Karmiloff-Smith (1985) developed a three-stage developmental problem-solving model to capture and explain children's reference and discourse construction strategies for narrating from a picture stimulus. The first level reveals a “procedural stage,” which in studies of narrative was manifest in productions by children aged 4–5 years. Theirs were lexically rich stories, but with frequent deictic use of pronouns to refer to story characters irrespective of discourse function, showing “bottom-up” reliance on the stimulus for narrative organization. Here, rich narrative content accompanied ambiguous reference to characters, through a “here-and-now” reliance on pronominal expressions. At the next, the meta-procedural stage, children aged 6–8 years were increasingly reliant on “top-down” processes, as the child used the main character, the “thematic subject” to construct the discourse and lead the narrative content, sometimes with the omission of other characters and the events in which they appear. The subject slot and pronominalisation was reserved for this referent, irrespective of discourse function (maintenance or switch). Karmiloff-Smith called this a “thematic subject constraint” (1985, p. 71). In the third stage, children's use of pronouns and other referring expressions is more flexible and these are deployed to globally organize the referencing task more uniformly, with rich lexicon and story detail.

Karmiloff-Smith's work spurred others to explore discourse strategy, as it provides a way to examine developmentally the dynamic interaction between a speaker's cognitive and perceptual response to, and formulation of, narrative content and the form–function pairings for reference realization in the construction of cohesive and coherent discourse. In his analysis of narrations by German-speaking children, Bamberg (1987) identified three further strategies, in addition to the *thematic strategy*. These were:

- an *anaphoric strategy* “which reserves the reference switching function for nominals, and the reference maintaining function for pronouns;
- a *locally contrasting strategy*, that shows no clear preference toward a clear separation of a form-function pairing and
- a *nominal strategy* which avoids the use of pronouns and just makes use of reference switching devices in a local and descriptive narration of each individual picture” (p. 93).

Bamberg elicited narratives with the picture stimulus “Frog, where are you?” (Mayer, 1969), used in wide range of studies (Berman and Slobin, 1994b; Wigglesworth, 1997; Strömqvist and Verhoeven, 2004), including the present study. It is important to note that the procedure in Bamberg's study involved the children hearing an adult narration of the story before narrating the story themselves, and this may have made the task less demanding, particularly for younger children. The study found that the anaphoric strategy was used most widely by 10-year-olds. The thematic strategy and the locally contrasting strategy were used by 4- and 6-year olds. The nominal strategy was used by a small number of 4-year-olds only.

Taking the matter further, Wigglesworth (1997) drew on this and other studies (Hemphill et al., 1991) to carry out fine-grained analyses of individual discourse strategies of frog story narrations by Australian English speaking children aged 4-, 6-, 8-, and 10-years, with 20 stories in each age group. The story was divided into four segments, and each segment in the individual narrations was analyzed to explore strategy use, as a factor of complexity involved in the referencing task in each segment. Wigglesworth devised criteria for identifying strategies, based on the percentage of pronominal expressions used for switching reference to the main characters in the story, along with the percentage of nominals used to refer to these characters overall (1997, p. 288), discussed further below. Following from Karmiloff-Smith and Bamberg, Wigglesworth (1997, p. 291) classified segments as follows:

- *thematic* when switches of reference and reference maintenance to the main characters were overwhelmingly pronominal,
- *partial thematic* when a high proportion of switches to the main characters were pronominal.
- *anaphoric* when switches of reference to the main characters were nominal and maintenance was pronominal.
- *nominal* when nominal references were clearly preferred regardless of function.
- *no (apparent) strategy* when “form/function pairings appeared to be randomly distributed.” This was necessary for a small set of productions by the youngest group could not be clearly classified.

The anaphoric and thematic strategies provide more *global* means for discourse-wide organization, while the nominal and no-apparent strategy serve to organize discourse *locally*, at the level of the clause or page.

The study showed considerable variability across the age groups. Importantly, children at different ages were vulnerable to the changing demands of the story prompt, with younger children switching between strategies, particularly in referentially complex segments of the long story, while 10-year-olds were less vulnerable to the changing referential demands in the story. Overall, 4- and 6-year-olds used strategies, with the partial thematic and thematic strategy most frequent, but with strategy shifts to increasingly local strategies. Some 8-year-olds used the anaphoric strategy, but overall a range of strategies were used. The 10-year-olds showed increasing use of the anaphoric strategy, while the adult narrations were overwhelmingly anaphoric.

From the analysis of discourse strategies Wigglesworth posited five stages of discourse organization aligned to the strategies, from local, where reference is organized at the level of the clause and page, to increasingly global, where cohesive reference management takes place at the level of the segmental, the simplified narrative (in terms of lexical richness, story content) and finally, cohesive and coherent narrative (1997, p. 305).

The present study builds on this previous research and analyses discourse strategies to explore narrative development among Wumpurrarni English speaking children.

## The present study

Narrations by 40 Wumpurrarni English speaking children, aged between 5 and 14 years are analyzed. Eight narrations by adult speakers provided a model of mature production. While the initial design was not a comparison of home and school varieties, some of the children spoke in a style associated with the ways that children are required to speak at school. This created the opportunity for a partial comparison. Thus, the study addresses the following research questions:

1. What developmental trajectory can be observed for Wumpurrarni English speaking children with respect to discourse development?
2. How can these be related to previous studies?
3. Can differences in reference and discourse strategy be seen in a comparison of the small set of school variety (SV) and home variety (HV) narrations be identified? And if so, what are the implications of this for education assessment and delivery?
4. Can the analyses extend understandings of the developmental mechanisms involved in discourse development?

Investigating language development in a small and dynamic language setting poses distinct challenges and insights. There are few descriptions of the kind of complex multi-code linguistic situations similar to the context for this study. Recent examples include (Sandefur, 1979; Hudson, 1983; Meakins, 2008; O'shannessy, 2008), with some research on the discourse pragmatics of such varieties (Graber, 1987; Disbray, 2008; Nicholls, 2011, unpublished manuscript; Meakins and O'shannessy, 2010; Sharifian, 2010). A further challenge is the high level of inter- and intra-speaker variation. The “target” is difficult to pinpoint and the mix of features that will constitute input to children is difficult to predict, as is true of language development studies in other contact settings (Youssef, 1991, 2005; Carrington, 1995; O'shannessy, 2009; Dixon, 2013).

### The bivarietial language setting: wumpurrarni english and school english

An estimated 25,000 Aboriginal people across Australia speak English-lexified creole languages (Australian Institute of Aboriginal Torres Strait Islander Studies in association with the Federation of Aboriginal Torres Strait Islander Languages, 2005). Wumpurrarni English is one such code. It is spoken by around 1000 people in the small remote township of Tennant Creek, in northern Central Australia, on Warumungu country. Wumpurrarni English shares some features with Warumungu (Disbray and Simpson, 2005; Morrison and Disbray, 2008) and with other contact varieties spoken across remote Australia, such as Roper River Kriol, spoken to the north (Harris, 1993). However, it is linguistically and socio-linguistically distinct from Kriol.

An English-lexified variety, it shares many features with Standard Australian English, with which speakers are in close and constant contact. This is particularly true of children, as Australian English is the medium of instruction in school. There is no recognition of, or learning support for, the students as learners of English as an Additional Language/Dialect. However, all children in the study are emergent multi-dialectal/-lingual language users (Morrison and Disbray, 2008). As in other settings, contact varieties in Australia tend to have low-status, and their role in speakers' repertoires, language development or potential role in education is little recognized (Siegel, 2006; Malcolm, 2011; Sellwood and Angelo, 2012; Dixon, 2013). Wumpurrarni English does not have a literate tradition and the orthography used in the present study is an adaptation of the Kriol orthography (Lee, 2002).

### Wumpurrarni english styles

Wumpurrarni English styles vary from basilectal styles referred to by speakers as “heavy” to acrolectal or “light” styles. Characterizations of these styles are not categorical, but are based on a combination of features and their frequency in a given stretch of speech. Most speakers have access to a repertoire of styles and can shift between these depending on the situation, and this is an important aspect of children's language development in this setting. The range of an individual's repertoire is influenced by factors such as residence and employment patterns, and in the case of children, residence and employment patterns of parents and care givers. The following three extracts from the frog story data for this study are provided to give some sense of this variation as this is has bearing on the developmental study of reference which follows. Note interlinear glossing is used in this section only, to illustrate morphological features of Wumpurrarni English^1^.

Example 1 by an 8 year old child (C8.9) shows a “heavy” style of Wumpurrarni English. Note the age of the speaker is encoded in the first digit of the text identification code.

1.

- Dat  lil      boi   an       dat     kunapa   slip  na
  Det  little  boy   conj   Det    dog        sleep Dis
  *The little boy and the dog sleep*.
- An   frokfrok     i     bin     ran-awei.
  con   frog          3S   Pst     run-away
  *The frog it escaped*.
- Dubala      bin    gid-ap na.
  3Du          Pst     get-up Dis
  *The two of them got up*.
- Dei   bin   luk,   nading na.
  3Pl   Pst   look   nothing Dis
  *He looked, but in vain*.
- Im an       dat kunapa     bin gid-ap     fom bed.
  3S Conj   Det dog         Pst get-up     Prep bed
  *He and the dog got up out of bed*.
- I      bin       pul-im-an           im-kayi    but      na.
  3S   Pst      pull-Trans-on     3S-Poss    boot    Dis
  *He pulled on his boots*.

In this extract there are some features from Warumungu, *kunapa* “dog” and the possessive marker –*kayi* (lines 1.a, f). Bare nouns generally occur in preposition phrases (line 1.e) and alternate with determiner + noun phrases in argument position (line 1. a, b). Transitive marking (verb + *im*, line 1.f) and general past tense marker *bin* “Past” from Kriol (lines 1.b-f) are used consistently, reflective of this style.

The “lighter” style in the next extract, by another 8-year-old (C8.7) shares some of these features, however with variable past tense marking; the use of *bin* (2.c, e) alternates with past tense marked on verbs (2.a, b) and unmarked verbs (2.d). The determiner form is *da* rather than *dat*, from “the” and “that” in English respectively, and occurs with all nouns in argument positions, and determiner + noun alternates with a bare noun in preposition phrases (2.b, e). Possessive determiners *is* “his” and *ma* “my” occur (2.a, d), rather than the pronoun + possessive suffix *imkayi*.

2.

- Da  lidl      boi  and     is           dog    went    to    slip    den.
  Det little   boy  Conj   3SPoss  dog    go-Pst to   sleep   then
  *The little boy and his dog went to sleep then*.
- An      da     frog    krip-ed      out     fom    da     jar.
  Conj   Det   frog   creep-Pst   out    Prep    Det   jar
  *And the frog crept out of the jar*.
- I     bin    git-ap    fom    slip.
  3S  Pst   get-up    Prep    sleep
  *He woke up*.
- I       tok,   "eh,    weya-s   ma        frog?
  3S   say     Dis    where is 1Poss   frog
  He says, “hey, where's my frog?”
- I      bin   luk     evriweya,       anda     shu-s.
  3S  Pst   Look   everywhere   under   shoe-Pl
  *He looked everywhere, under the shoes*.

Twelve stories in the data set were narrated in what is referred to in this paper as school variety (SV). These texts have been identified through the use the English rather than the Wumpurrarni English verbal system, though Wumpurrarni English features occur in all narrations (e.g., the preposition *gad* “with” in line 3.f.). The extract in example 3 from (C8.8) is one such text.

3.
  - Scare crow make the little boy fall down fom the tree.
  - And that little poor thing duck.
  - And dat little papi traina go for im up there.
  - And the little boy hanged on for that thing.
  - And the little boy reckon dis was the tree.
  - And the little boy was going gad that thing.

The use of Standard Australian English orthography obscures non-standard phonology in this production, but captures the more English style.

### Reference in wumpurrarni english

Referring expressions in discourse can be thought of as a hierarchy, according to the level of accessibility or predictability of the referent, with a general principle in which less predictable information will be given more coding material (Givón, 1983, 1990). In English for instance, forms such as referential indefinite nouns mark the least accessible/predictable referents, definite nouns the middle ground, and realized and zero pronouns, highly recoverable referents. These principles hold largely for Wumpurrarni English. However, some further features should be highlighted.

With respect to lexical nouns, there is no grammaticised marking of newness versus givenness, as in indefinite vs. definite articles in Standard English. Lexical nouns may be bare or include a determiner, as in the examples above, but this is a factor of stylistic variation rather than grammatical principles. However, discourse prominence does provide some account for this alternation. In Bruyn's (1994, p. 264) discussion of the development of articles from demonstratives and numerals in Creole languages, she writes:

[A]s long as [determiners] have not become plain articles, they alternate with zero. And as long as the overt determiners are not used categorically, they keep a stronger value, such as demonstrative or emphatic. If there is no reason to use [a demonstrative/article form] to give emphasis, to single out a referent, or for other purposes, the bare noun will suffice.

This alternation is evident in Wumpurrarni English, particularly in heavier styles.

A further feature of reference in Wumpurrarni English is repetition (Disbray, 2009), common in discourse in contact languages (Meakins and O'shannessy, 2010) and Australian Aboriginal languages more generally (Bavin, 2000; Walsh, 2006). In Wumpurrarni English, full lexical nouns may occur as “emphatic subject chains,” in which subsequent mentions are not reduced to an anaphoric pronoun, or elided, as in (4.a-b) below. Using this structure, speakers “build up” a story, repeating elements of a clause, including the full lexical referent and adding detail in a set of chained clauses, as in the following by (Adult 5):

4.

- Dat lidlboi   bin klaim   nanga   ston-kana,   na olabat.
  Det boy       Pst climb    Loc      stone-Loc   Dis all around
  *The little boy climbed up all the rocks*.
- An      dat lidlboi    klaim    Rait top na,      na hil-kana.
  Conj   Det boy      climb     Right top Dis   Loc hill-Loc
  *And the little boy climbed right to the top of the hill*.

The use of full noun phrases for emphatic discourse purposes is an important feature of Wumpurrarni English discourse pragmatics and is incorporated into the model of discourse strategies below. Often, though not always, repetitions in subject chains include left dislocated structures, which consist of a full lexical noun with a resumptive pronoun. This structure is used for initial introductions, and to re-introduce and maintain reference referents in discourse. Givón has suggested that functionally left dislocation is “typically a device to mark topical referents, most commonly definite and anaphoric ones, that have been out of the focus of attention for a while and are being brought back into the discourse” (Givón, 2001, p. 265). This is the case in line 1.b above, in which the frog has already been introduced and attention has shifted to the boy and the dog. As frog's unexpected and plot-propelling escape is detailed, reference is made with a left dislocated structure. This use of nominals to maintain reference and mark salience and emphasis is a point of difference between Wumpurrarni English and Standard Australian English discourse pragmatics. In one 8-year-old speaker's frog story narration (C8.7), this structure signals and emphasizes the unexpected first appearance of the bird:

5.

- Bird   i   bin   kam   at       fo       im.
  bird   3S  Pst  come  out     Prep 3SObj
  *A bird flew out at him*.

Some final comments regarding pronominal forms in Wumpurrarni English are worth noting. First, the third person singular subject form is most commonly realized as *i* from English “he,” and does not distinguish gender or animacy of the referent. In addition, Wumpurrarni English allows zero subject and object realization in a wider range of contexts than English.

## Methods

### Participants

Narrations by 48 participants are analyzed in this cross-sectional study. Most participants also took take part in a longitudinal study of language development, the Aboriginal Child Language Acquisition project^2^ (Disbray and Wigglesworth, 2008; Simpson and Wigglesworth, 2008). The corpus generated from the longitudinal study has informed the language description of Wumpurrarni English, and so provided information on the repertoires of individual speakers. Table 1 shows the number of participants in each age group.

**Table 1:** **Participants and Ages**.

**Age groups**	**5–6 years, *n* = 10**	**8 years, *n* = 10**	**10 years, *n* = 10**	**12 years, *n* = 10**	**Adults, *n* = 8**
Mean Age	5;10	8;5	10;4	12;11	/
Age Range	5;4–6;	8;0–8;10	10;3–10;10	12;4–14;1	22–55

### Materials

The narrations were elicited using the stimulus, “Frog, where are you?” (Mayer, 1969). This textless picture book provides a problem-resolution framework, in which a boy and his dog take part in a number of search attempts for their escaped frog, encountering five secondary characters before the plot resolution (Trabasso and Rodkin, 1994). It has been used widely in developmental studies of reference in narrative in one language and cross-linguistically, (Berman and Slobin, 1994b; Strömqvist and Verhoeven, 2004), including two Indigenous Australian languages, Arrernte and Warlpiri (Bavin, 2000). The story is quite long with 24 pictures over 15 pages. For the analysis, the pages were grouped into four segments. The pictures, pages and segments are described in Supplementary Materials.

### Procedure

Participants viewed the picture and then were asked by the research assistant in WE to talk as they do at home, “Wumpurrarni way” and to tell the whole story. The researcher sat beside a video camera on a tripod, at a distance of approximately 1.2 m from the speaker, preventing shared visual access to the book. The small book format required both hands, which reduced pointing and encouraged full verbal accounts. The length of the productions varied, as displayed in Table 2. The data were transcribed using the CHAT format of the Child Language Data Exchange System (CHILDES) (MacWhinney, 2000), with each line representing a communication unit (C-unit). An utterance is transcribed as a c-unit when its syntax is complete for the context of talk, it has a terminal intonation contour and/or is followed by more than 2 s of silence. The number of C- (communication) units was calculated for each speaker, the mean number and range for each group are set out in Table 2. The mean number of C-units indicates an increase with age, and the range is wide.

**Table 2:** **Mean and range in number of C-units by age**.

**Age**	**6**	**8**	**10**	**12**	**A**
Mean number of C-units	32.6	37.7	47.9	45.3	78.1
Range	22–52	26–44	29–59	29–75	41–94

### Operationalization of factors for analysis

#### The demands of the story segments

To investigate individual responses to the changing referential load in the story, each narration was divided into four segments. The cognitive processing demands of each was determined by the number and activity of characters. In segment one, following the introduction of the central three characters (boy, dog, and frog), at least one character acts alone throughout the segment. In the second segment the activity moves outside, and the boy and the dog again undertake different activities. Additionally, a number of secondary characters appear and actively interact with one or other of the two main protagonists. In the third segment attention is focused on the boy. The dog does not appear in the first pages of this segment, and once it does, the boy remains in the foreground. The final segment is again marked by a change of environment with the boy and the dog sitting in a pond. From this point they largely act together, finding a family of frogs, and finally leaving with one of the baby frogs.

It was hypothesized that the processing requirement of each segment varies in terms of the number of characters and the activities they perform. Initially, the activities of the frog, critical to the storyline, must be detailed. However, once it is gone, the boy and the dog remain and undertake different activities. From this point, there are a number of ways to narrate the events. For instance, the focus may be on one character in particular, with the activities of the other ignored. Both characters may be referred to plurally, detailing only one set of activities, or the activities of both may be separately described. Thus, there is a dynamic interaction between what is depicted in the book and how the speaker describes the events. Nonetheless, the processing load of the second segment is greater than that of the first and subsequent segments, because new characters must be introduced and their active interactions with the main protagonists detailed. In contrast, the third segment begins with a picture of the boy alone. From this point on his activities are critical to the storyline. The single focus in the segment represents a simpler task cognitively. This final segment task demands are also cognitively simpler as the boy and dog act together for the most part. The referring task may again be reduced to detailing a single set of events.

In examining the narrative at the segmental level, attention is paid not only to form-function pairings and discourse strategy, but also the interaction of these with narrative content and story elements, crucial to comprehensive insight into children's narrative development (Mäkinen et al., 2014, p. 27).

#### Coding at clause level

The coding system at the clause level is driven by the local “recoverability” of the referent, determined most importantly by the newness of the referent, the fullness of the form, and the identity of the referent. In CHAT each clause was assigned a “reference” tier and a numerical code was given to each animate referent along three dimensions:

1. Protagonist: the boy, dog, boy, and the dog acting together, frog, and other (covering the five secondary characters) were coded with a numerical code;
2. Function: A set of abbreviations were developed to code first mention, same or different referent; and grammatical role–subject and object;
3. Linguistic form–nominal vs. prominal form.

Supplementary Materials explains the operationalization of this coding. Once all referring expressions for the identified protagonists in each transcript were coded and checked, the program FREQ in CLAN was used to compile all instances of all coding combinations on the reference tier of each narration. Results were exported immediately into Excel spreadsheets and sorted in various ways, before calculations were made. Note, hesitations are marked [/], retraces [- - -], and inaudible material [xx].

#### Linguistic form–nominal and pronominal forms

The children could refer to the characters in their narratives using the main set of referential forms shown in Table 3.

**Table 3:** **Nominal and Pronominal forms**.

**Nominal forms**			**Pronominal forms**	
Proper noun	*Princess*		Personal pronouns,	*i* “he/she/it”
Bare noun	*dog*		subject and object	*im* “him, her, it”
Bare noun + pronoun	*frok i*		(singular)	
Determiner + noun	*da boi/dat boi*		Personal pronouns	*dubala* “they 2”
Determiner + noun + pronoun	*dat boi i*		(dual and dual/plural)	damob, dei “they”
Possessive pronoun + noun	*imkayi frokfrok*		Possessive pronouns	*imkayi, is* “his, hers its”
				*damob-kayi* “theirs”

#### Defining the discourse strategies

Models from previous studies (Karmiloff-Smith, 1983; Bamberg, 1987; Wigglesworth, 1997) were adapted to identify five discourse strategies. The *thematic strategy* involves switches of reference and reference maintenance to the main characters overwhelmingly with a pronominal expression. The *anaphoric strategy* involves reference to the main characters with a nominal and maintenance with a pronominal. To accommodate the proportion of expressions with full lexical nouns in continuing reference contexts for emphatic purposes in Wumpurrarni English storytelling, the percentage of nouns overall in the anaphoric strategy is higher than in Wigglesworth's model devised for Standard English (Wigglesworth, 1997, pp. 287–288), which set the percentage of nouns overall at <50%.

Segments were classified as *nominal* where nominal references were clearly preferred regardless of function. Two additional adaptations were made. A *pronominal strategy* was added to capture some narrations in which few nominals appeared at all. And rather than a *partial thematic strategy* as proposed by Wigglesworth (1997), the current study identified a l*ocal anaphoric strategy*, which involves stories with a high proportion of nominals for reference maintenance and switch and some use of anaphoric pronouns for reference to the main character(s).

Preliminary analysis showed the boy or the boy and dog acting together to be the predominant thematic subjects. Thus, the criteria for discourse strategies focus on these character sets. Two measures were devised to assign the strategies: the percentage of pronouns to switch reference to the boy or boy and dog, and percentage of nominals overall for these characters. As an additional count was required to distinguish the thematic from the pronominal strategy, the percentage of nouns overall was used. The criteria for each strategy are set out in Table 4.

**Table 4:** **Criteria for strategy groups**.

**Strategy**	**Pronouns for switch reference to boy and/or boy&dog (%)**	**Nouns overall for reference to boy and/or boy&dog (%)**
Pronominal	>80	<20^*^
Nominal	0–20	>60^*^
Local anaphoric	20–40	>60
Thematic	>70	<50
Anaphoric	0–40	<60

* *nouns overall*.

## Results

Previous studies using a model of discourse strategies have shown that developing ability to organize and produce narrative is revealed through moves from data-driven management with local organization (at the level of the clause or page), to more global discourse organization (establishing and maintaining cohesion across stretches of discourse and discourse-wide) (Karmiloff-Smith, 1981, 1983; Bamberg, 1987; Wigglesworth, 1997). The coding procedure was designed to identify local referencing discourse strategies (pronominal, nominal and local anaphoric) and global discourse strategies (anaphoric and thematic), based on the ways that speakers maintained and switched reference to the two main characters acting independently and together acting as a pair. As the processing task of the various segments varied in complexity, the discourse strategy for each segment was identified. This showed whether the narrator used the same strategy throughout, or shifted strategy at some point in the narration. It was predicted that younger speakers might be more vulnerable to the changing task complexity than older children (Wigglesworth, 1997; Berman, 2004).

In addition to the variables of age and task complexity, the current study explores the extent to which productions by speakers using home variety (HV) might differ to those using school variety (SV), with respect to reference and discourse strategy, and as a consequence, global organization.

### Overview of results by age and variety

**Figure 1:**
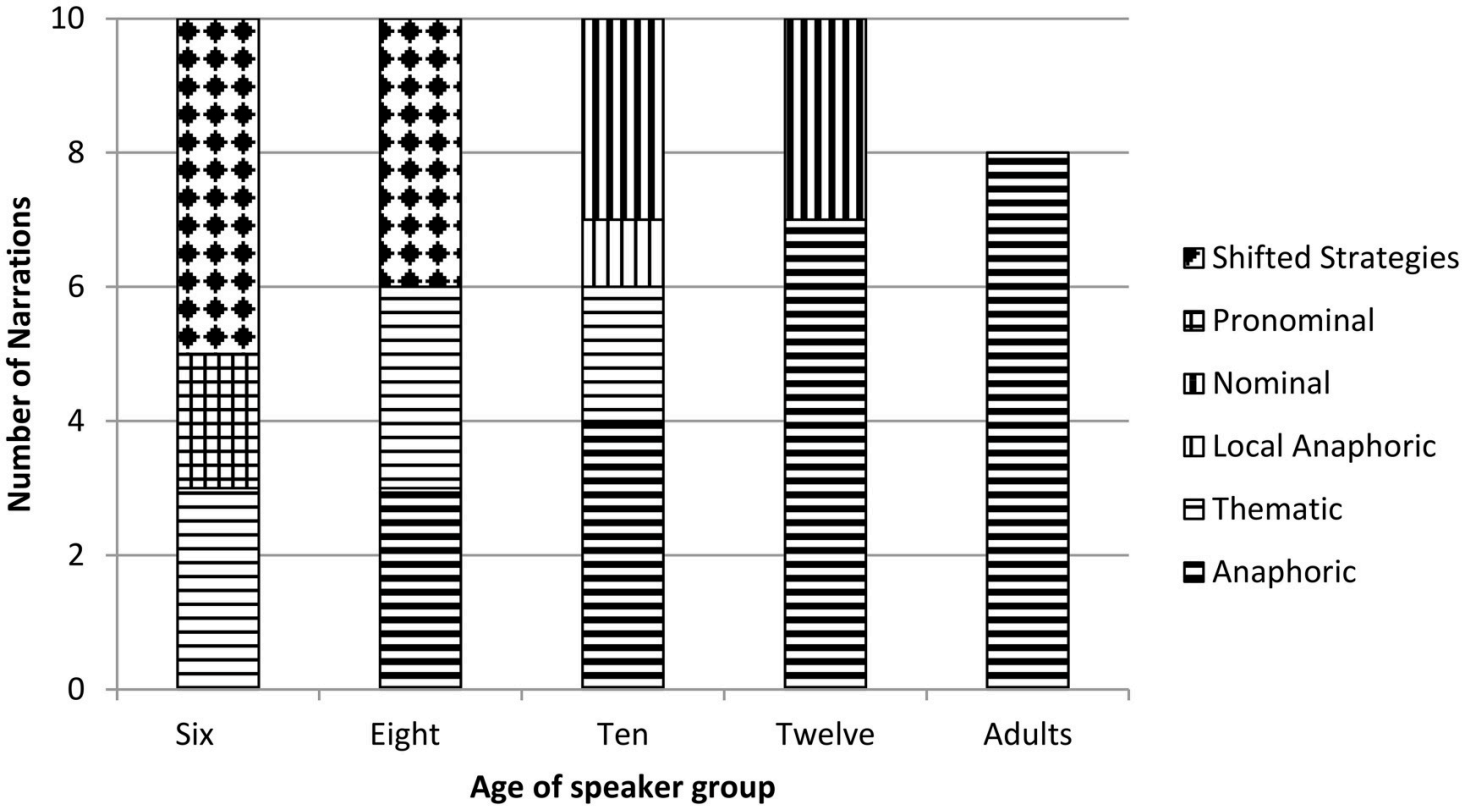
**Discourse strategy by age: all stories**.

**Figure 2:**
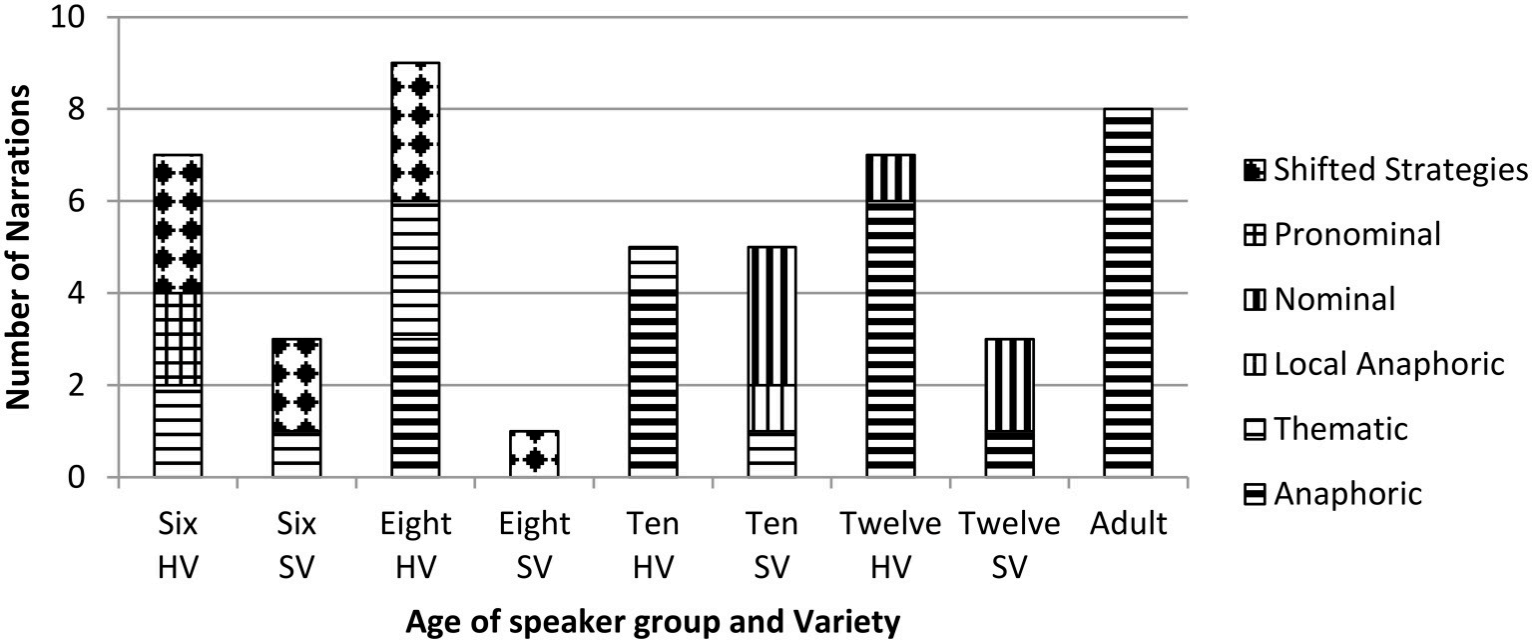
**Discourse Strategy by age, Home Variety (HV), and School Variety (SV)**.

Before turning to the results for discourse strategy by age (Figure 1) and discourse strategy by age and language (HV and SV) (Figure 2), Table 5 shows the length of narrations by setting out the mean number of C-units by age, and by language.

**Table 5:** **Mean and range in number of C-units by age and Home Variety (HV) and School Variety (SV)**.

**Age**	**6**	**8**	**10**	**12**	**A**
Mean number of C-units (All)	32.6	37.7	47.9	45.3	78.1
Range [No. of narrations]	22–52 [10]	26–44 [10]	19–59 [10]	29–75 [10]	41–94 [8]
**HOME VARIETY**					
Mean number of C-units	27.5	38.55	61.2	49.14	
Range [No. of narrations]	22–38 [7]	26–44 [9]	44–54 [5]	30–75 [7]	
**SCHOOL VARIETY**					
Mean number of C-units	43.33		34.6	36.3	
Range [No. of narrations]	39–52 [3]	34 [1]	19–49 [5]	29–45 [3]	

The mean numbers for all narrations indicates an increase in story length with age, with the exception of the 12-year-olds. The mean for these stories is slightly less than in the mean for 10-year-olds. Among these two groups, the length of stories told in HV is longer than in SV, but the reverse is true among the 6-year-olds. With just one eight-year-old SV text, no statement can be made. Matters of language variety are pursued below.

Results for the analysis of discourse strategy in all narrations by age group, are laid out in Figure 1. Narrations in which more than one strategy was used are counted as “Shifted Strategy” (shown in the checkerboard pattern).

Age-related patterns are summarized thus. The use of the globally organizing strategies increases with age. All adults and most 12-year olds organized their narratives with an anaphoric strategy, as did four 10- and 3-eight-year olds. This strategy was not used in the narratives of any 6-year olds. The other global strategy, the thematic strategy, was used by speakers in the three younger speaker groups only. The pronominal strategy appeared in narrations by 6-year-olds only. Only speakers in the two youngest groups shifted between discourse strategies.

One ten-year-old speaker used the local anaphoric strategy to organize his narrative. The final result of note is the use of the nominal strategy throughout by 10- and 12-year-olds.

Figure 2 shows the results for discourse strategy by age (child groups only) and by variety used in the narration. School variety was used in a small subset of stories by three-six-year olds, one eight-year old, five ten-year olds, and four of the twelve-year olds. However, there are notable trends here, which intersect with age related findings.

Three six- year-olds narrated in SV. One used a thematic strategy, while two shifted strategies, along with the only 8-year-old, who narrated in SV. This appears to be in line with the broader developmental findings for these age groups, and is further discussed below.

The results for the older groups, on the other hand, show a correlation between the use of HV and globally organizing strategies, and the use of SV and locally organizing strategies, the nominal strategy and the local anaphoric strategy, and as shown above, HV narrations were longer than SV narrations among these groups.

The following discussion first considers findings for the HV then findings for the SV productions.

### Discussion of results by age-home variety

#### Adult narrations

The adult texts were organized with an anaphoric strategy, with secondary characters, changes in locations and connections between events clearly expressed. The average length was greater than any of the child groups (Table 5). Six of the eight were over 80 clauses, the other two, 50 and 41 clauses. They included instances of subject chaining, repetitions of subject noun phrases across adjacent clauses (example 4 above), and their use provided justification for adapting the anaphoric strategy descriptor, allowing for a higher percentage of reference maintenance with a full nominal (Wigglesworth, 1997). This feature of Wumpurrarni English contrasts with the narrative pragmatics of the speakers second dialect, Standard Australian English. The adult texts provide a model for the HV narrations.

#### Six-year-olds

Seven of the ten six-year-olds' narrations are in HV. The individual strategy patterns among this group overall are more varied than among the other groups, and most simplified orienting, plot and character information. Two used the least mature strategy, the pronominal strategy. In previous studies such “here-and-now” narratives are commonly found among 4-year-olds and also some 6-year-olds (Bamberg, 1987; Wigglesworth, 1997; Bavin, 2000; Severing and Verhoeven, 2001). In the instances in the current study, the speakers used very few nouns overall and relied heavily on the stimulus to organize the narrative, rather than on linguistic means of creating reference cohesion. The listener thus requires knowledge of the pictures to follow the story. Lines 6. a-g in example 6 illustrate the *pronominal strategy*:

6.

- Papi  jamp  na  hed  na  smashim.
  *The dog jumps on his head smashing (it)*.
- An  i  bin  singat.  (i = the boy)
  *And he/it calls out*.
- An  i  bin  woof.  (i = the dog)
  *And he/it barks*.
- An  i  bin  foldan.
  *And he/it fell down*.  (i = the dog)
- I   bin  go  ran.
  *He/it went running*.  (i = the dog/boy?)
- Bi  bin  jeisimbat  dem.
  *Some bees went chasing after them*.
- An  i  bin  klaindap  na da ding.  (i = the boy)
  *And he/it climbed up on the thing*.

The pronominal strategy also appeared in the narration of one child who shifted strategies; between a pronominal strategy in two segments, then a thematic strategy for the last two. The thematic strategy was also used in the two further narrations with strategy shifts. They began with a thematic strategy and changed in response to the complex second segment to locally organizing strategies (local anaphoric or nominal), illustrating a bottom-up dynamic interaction with the prompt. This local organization was also evident in the frequent use of left dislocated structures to signal contrast, as they shifted between characters and depicted one individual event after another. In doing so, they did not generate coherence to the story-line overall. This is exemplified in example 7, (C6.4)'s narration:

7. (Page 5, Segment 2)

- An dei bin singat.
  *And they called out*.
  (Page 6A, Segment 3)
- An dat boi i bin luk ina hol. *And the boy, he looked in the hole*.
- An dat papi i bin luk dat bi an [xx].
  *And the dog, it looked at the bee and* x.
  (Page 7)
- An dat boi i bin luk weya dat tri.
  *And the boy, he looked in the tree*.
  (Page 8)
- An den, dat boi i bin foldan.
  *And then the boy he fell down*.
  (Page 9, Segment 3)
- I bin go weya dat rok.
  *He went to the rock*.

Both retained the local anaphoric strategy or nominal for the third segment and then returned to a thematic strategy for the fourth segment, pronominalising joint reference to the boy and dog, as they appeared together once more.

The two remaining narrations in this set were thematic, with the referencing task managed in a unified way across the narrative. These speakers frequently selected the boy and dog together as the thematic subject.

Overall, the considerable variability in the narrations by the by 6-year-olds show a high level of flux on the developmental trajectory from locally organized to more globally organized discourse. Most simplified the story content and referencing task, and relied heavily on the pictures to structure their stories. However, task complexity clearly posed challenges to most speakers in this group, with just three maintaining the same strategy across the first and second segments, and the others using a local strategy for this, either for this segment or throughout.

#### Eight year olds

Nine of the ten eight-year-olds' narrations are in HV. Five speakers used a global strategy and maintained this throughout; three with an anaphoric, and two with the thematic strategy. Overall, there was a clear preference for a thematic strategy among this age group. Of the six who began with it, two maintained it and three switched strategy to either the local anaphoric or the nominal strategy. All shifted back to a global strategy, an anaphoric or thematic strategy, in the last segments. The production by (C8.7) provides an example of a thematic narration, with the boy selected as thematic subject, as switches to the boy are made with a pronoun, rather than full lexical noun.

8. [Page 4B]

- Dat frokfrok nat na dat ding.
  *The frog wasn't in the thing (jar)*.
- I bin luk na but, nothing.
  He looked in the boot, in vain. (i=the boy)
- An papi trai smal fo im, inti?
  And the dog sniffs around for it, doesn't he?
- An i bin smash dat ding.
  *And it smashed the thing (jar)*.
  [Page 5]
- An i bin singat fo frokfrok. (i = the boy)
  *And he calls out for the frog*.
- An bi i bin kamat.
  *And bees come out*.
- Papi bin ran.
  *The dog runs*.
  [Page 6A&B]
- I bin lukabat fo frokfrok. (i = the boy)
  *He looks around for the frog*.
- I bin singat.
  *He calls out*.

Although, all 8-year-olds used a global strategy for part or all of their production, in comparison to the age older groups, these were simpler stories, with less narrative content and higher levels of omission of secondary characters. As a result the 8-year-olds' narrations were on average shorter than the age groups above (Table 2). Indeed, the complications involving the secondary characters in the second segment were either very brief and undeveloped or omitted altogether in most productions, true also among other speakers of the same age (Wigglesworth, 1997). Further, in most stories causal relationships between events were often not made explicit, in this anaphoric narration by (C8.9) shown in example 9:

9. (Page 7)

- Lidlboi bin klainingap na tri top,
  *The boy climbed to the top of the tree*,
- Ø luk nanga na hol.
  *and looked in the hole*.
  (Page 8)
- An dat jukjuk bin fraitenim dat [%kunap] [/] lidlboi.
  *And the bird frightened the boy*.
- An dem bis jeisimbat dat kunapa.
  *And the bees chased the dog*.
  (Page 9 A&B)
- Dat lidlboi klaimingap na rok top.
  *The boy was climbing on top of the rock*.
- I klainap na an i bin singinatbat na.
  *He climbs up and he calls out*.

The overall pattern in this age group is one of increasing ability to globally organize a simplified narrative, with two means of simplifying the task; by drawing on a thematic strategy to manage the referencing task and/or by simplifying the story plot through omission of story detail. In addition, speakers in this age group were more sensitive to the changing demands of the prompt than the older groups, with four switching strategies for the second and third segments, and all simplifying the story line at this point. However, the use of a global strategy by all speakers for at least part of the narration and among this age group showed increasing top-down discourse management, where child speakers are more able to co-ordinate the various tasks involved with narration, in line with findings by Mäkinen et al. (2014) and Shapiro and Hudson (1997).

#### Ten and twelve-year-olds

Five of the ten stories by 10-year-olds and seven by 12-year-olds were narrated in HV. All but one speaker used a global organizing strategy throughout, overwhelmingly the anaphoric strategy. These stories were detailed and unaffected by the changing demands of the second segment, as shown in example 10 by (C12.8).

10. (Page 4)

- Papi and dat lidl boi stil luk, singinat.
  *The dog and the boy keep searching, calling out*.
  (Page 5)
- Dei bin go langa bush na an singatbat.
  *They went into the bush and keep calling out*.
- Dei bin siyim bihaiv na.
  *They saw a bee hive*.
- Ola bimob musterin.
  *All the bees are swarming*.
- Ola dakwan tri du dei bin luk.
  *They saw all of the dark trees*.
  (Page 6)
- An dat lidlboi bin singinat insaid na hol bigwan.
  *And the little boy called out into the big hole*.
- An dat kunapa I bin jamp langa dat bihaiv, dat hani-kayi, bi.
  *And the dog, he jumped at the bee hive, bees*.
- An lidl munyunyu bin kamat an baitim dat lidlboi pawumpawu na nos.
  *And the little mouse came out, and bit the little boy, poor thing, on the nose*.

These mature productions included Wumpurrarni English story telling elements, such as repetition through subject chaining (11. a-b), contrast and emphasis through left dislocated structures (11. b, d, f) and object fronting (11. f), shown in example 11, by (C10.5).

11. (Page 7)

- An dat kunapa bin trai itimbat bi.
  *And the dog was trying to hit the bees*.
- Dat kunapa i bin trai nokim im na nathan tingabi-kayi.
  *That dog was trying to knock down the stinger bees' hive*.
- But bi traina jeisimbat im.
  *But the bees tried to chase the dog*.
  (Page 8)
- An dat bird na i bin trai fraitenim dat lidlboi,
  *And the bird tried to frighten the little boy*,
- an I bin foldan.
  *And he fell down*.
- And dat kunapa, bi i bin jeisimbat.
  *And the dog, the bees chased*.
- An dat bird bin stil folarimbat dat lidlboi, da lidlboi na.
  *And the bird was still following the little boy, the little boy there*.

One narration was classified as nominal. It contained instances of anaphoric reference, however overall the proportion of reference maintenance with a nominal, both within and across page boundaries, was high in this relatively short narration (34 c-units). The speaker (12.4) appeared to slip in and out of a picture description mode, but he flagged this by prefacing a number of the clauses with *deya na* “and now.” The use of this conjunction, the anaphoric references and the use of joint reference, contributed continuity and coherence to the events in this story, unlike the stilted effect in example 7.

12. (Page 11)

- And den dat buliki bin pushim dat lidlboi an dat papi.
  *And then the cow pushed the boy and the dog*.
- And dei bin foldan na.
  *And they fell down*.
- And deya na, dei bin foldan ina woda na.
  *And there, they fell down into the water*.
- An deya na, dat papi bin smalim na.
  *And there, the dog sniffed*.
- An lidlboi lisin da na.
  *And the boy listened*.
- An lidlboi bin tok ‘duim kwait na’.
  *And the boy said, ‘Be quiet now’*.

Overall, the developmental pattern for the 10- and 12-year olds HV narrations showed the ability to globally organize full and detailed narrations, with mastery of Wumpurrarni English features such as repetition and elaboration and fronting, similar to the adult narrations.

#### Narrations in school variety

Examination of the narrations in SV allows preliminary exploration of the differences in performance in the two varieties. Stavans (2001) has discussed “trades and concessions” that multilinguals may make in juggling the conceptual information and the linguistic choices available, and it is argued here that in the present study, children performed such juggling, and that there is evidence that trades and concessions were made. Trades and concessions were evident in one of the few developmental studies involving a contact language, by Severing and Verhoeven (2001). They investigated narrative development among children aged between 5 and 12 years, who speak a creole, Papiamento, as their home language and learn Dutch in school, similar to findings in the current study. Overall they found that children's narratives became longer and more elaborate with age in both languages. However, the stories told in Papiamento were consistently longer than those told in Dutch.

Further, “indicators of monitoring,” such devices as corrections, slips of the tongue, repetitions, false and restarts starts, were higher in Dutch than Papiamentio, particularly among the groups aged 8 and 10 years (Severing and Verhoeven, 2001, p. 261). With respect to reference realization, there was clear evidence of a developmental trajectory of increasing ability to track reference and avoid ambiguity in both languages, however they found some differences in the strategies children use to manage the referencing task in their respective languages. Overall proficiency in this aspect of narrative construction was greater in Papiamento than in Dutch (Severing and Verhoeven, 2001, p. 273).

In the present study, three six-year-olds narrated in SV. All speak a light variety of Wumpurrarni English at home and so school variety is not a massive style shift for these capable bi-dialectal children. All constructed long narratives in relation to the age group (Table 5), and all used the thematic strategy for all (in one case) or part of their story. The child who told the longest story (55 C-units) began his narration with a thematic strategy, but like other children in this group, shifted strategies at episode 2, to a local anaphoric strategy, maintaining reference with a nominal within and across page boundaries in almost equal proportions. He switched reference frequently, in a similar manner to the HV group (example 7). He paid close attention to his code choice, carefully choosing words and forms in this elaborate story, as in example 13:

13. (Page 11)

- And da reindeer chase the boy.
- And i chucked the dog and the boy in the water.
- And the dog and the boy fall down swim the water.
- And they had a good swim.
- And the boy and the dog getting happy.
- And they hear something from that hole there.

Given the individual language repertoires of the children and the similarities between the SV and HV texts with respect to discourse strategy patterns observed at this age, it is not possible to draw conclusions with respect to the impact of code choice on narration by the 6- and 8-year-old speakers, who narrated in SV. However, attention to the 10- and 12-year-olds' productions is warranted.

The results for the SV texts by 10- and 12-year-olds showed that six were locally organized by a nominal or local anaphoric strategy, and two were globally organized, with anaphoric and thematic strategies. Like the nominal segments in the narrations of the two younger groups (examples 7 and 13), few links between continuing referents were made, as each new clause began with a full lexical noun. However, in contrast to the younger groups, these speakers did not use the nominal strategy in response to the task load of the second episode, but used this strategy throughout. In further contrast to the younger speakers, and speaker 12.4 (example 12), these 10- and 12-year-old speakers rarely made use of reference to the joint activities of the two characters, which would have provided opportunities to pronominalize, which Orsolini et al. (1996) have referred to as the use of “parallelisms” in the text. Overall, the six SV stories lacked local and global level text cohesion. In addition, their use of nominals for reference maintenance was not in subject chains or repetitions, and so does not appear to be a persistent first language/dialect influence on discourse organization (cf. Benazzo and Andorno, 2010). Finally, these texts also featured hesitations, retraces and self-corrections including a correction to content, as in example 14 by (C10.7).

14.
  - da boy [/], na da dog fit he [/ - - -] no her nik na [/ - - -] neck in da tin.
  - da dog in da tin can not yell.

This speaker self-corrected content (“the boy, no the dog”), grammatical form (“he, no her”) and pronunciation (“nik no, neck”). These “indicators of monitoring” were not present in the HV productions.

Speaker (C10.10) used a local anaphoric strategy and also retraced utterances, making self-corrections (15.f), evidence of his attention to code choice and accuracy. The story is lexically rich (for example, further in the story, the child said, “he heard croaking sounds from the log”), however, this speaker omitted some secondary characters and referred exclusively to the boy, simplifying both the reference task and the narrative content. He used pronouns to maintain reference at the level of the clause, but lexical nouns at page turns:

15. (Page 6A)
  - Lilboi look ina hole an i couldn't see.
    (Page 6B)
  - then a mouse popped out of the hole.
    (Page 7)
  - Da boy searched in da tree, Ø could'nt see da frog
    (Page 8)
  - Da boy fell down an Ø got up.
    (Page 9)
  - Da boy climbed up da rock and Ø called out “where are you frog?”
  - den da boy ran and Ø fall [/ - - -] fell in da woda.

It is proposed that attention to lexicon and accuracy in SV came at a cost, with the referencing task locally managed and simplified, and with its resultant impact on overall discourse coherence.

Finally, one 12-year-old SV narration was classified as anaphoric. This lexically rich and globally organized story was simplified in various ways, not characteristic of the HV stories by 10- and 12-year-olds. In the first segment no mention was made of the separate searches by the boy and dog. In the second segment the separate encounters were simplified by joint reference, and two of the secondary characters were omitted. In the third and fourth segments reference was almost exclusively to the boy, with little mention of the dog. The production lacked some story detail, but the search motif was carried through the repetition of the phrase “but they still couldn't find him”:

16. (Page 5)
  - They went looking for him outside,
  - but they still couldn't find him.
    (Page 6A)
  - They looked in the holes and the bee hives and up the tree.
    but there was no frog there.
  - They went looking for him in the forest,
  - but they still couldn't find him.
    (Page 6B)
  - They looked on top of the trees and down below,
  - but they still couldn't find him.
    (Page 7)
  - Once the dog hit the bee hive and made it fall.
  - Then the bee came down, it chased the dog.

This was the most fluent SV text in the set, demonstrating this bi-dialectal speaker's high level of English language competency. Yet the speaker simplified the task in ways characteristic of the younger age groups at the level of narrative content, and by extension, the referencing task. It is posited that this speaker made “trades and concessions,” masterfully juggling the bi-dialectal resources at her disposal.

## Discussion: developmental trajectories and mechanisms

Previous studies propose that use of specific strategies reveals developmental stages or levels of discourse management (Karmiloff-Smith, 1983; Bamberg, 1987), as referential form-function patterning shows moves from local level organization, clause and page, to larger segments, simple narrative, and finally globally organized, content-rich narrative (Wigglesworth, 1997).

The analysis found anaphorically organized narrations to be most mature, used in the HV stories by 10- and 12-year-olds and adults, confirming previous developmental studies. Further, attention to these narrations advances our understanding of Wumpurrarni English narrative pragmatics, but also confirm Berman's characterization of discourse as “a meeting ground of developing linguistic knowledge and general cognitive growth.” The anaphoric strategy captured narrations (or segments) in which speakers sometimes maintained reference nominally, repeating references to characters and other story details with chained subjects, building up, and emphasizing story elements at points, while also using pronouns to maintain reference within and across page boundaries. In doing so, speakers controlled the representation of events through the actions of characters in the broader narrative structure. Their choice of referring expression is guided by the need to disambiguate reference, attending to the listener's knowledge state, but not exclusively to link back to an antecedent expression. Rather, manipulation of reference foregrounds aspects of the story action, with nominal expressions contributing salience in an interaction between local cohesion and discourse coherence, as speakers exploit the pragmatic predictability of a referent. This narrative feature is an example of Wumpurrarni English “thinking for speaking” (cf. Slobin, 1996, 2001). In addition, it becomes clear that this global management of reference marks salience and organizes story content and, as the speaker attends on-line to both previous utterances and forward planning across the discourse, reveals an intersection of linguistic and cognitive performance.

The pattern for 6- and 8-year-olds showed a developing ability to manage reference, as children come to deploy linguistic devices flexibly and appropriately in a widening range of contexts (Karmiloff-Smith, 1992). Most of the 6- and 8-year-olds organized some or all of their stories with a thematic strategy, and some 8-year-olds, an anaphoric strategy. The thematic strategy similarly exploits the pragmatic salience across a segment/narrative, but this is limited to the main characters, and their salience is indexed by pronominal reference, rather than the more flexible and nuanced Wumpurrarni English anaphoric strategy. It is guided not only by presuppositions about the listener's knowledge state, but by the speaker's construction of the narrative scheme, managed “top-down” by reference to main characters (Karmiloff-Smith, 1985). The planning task is simplified as the subject slot is more prescribed than in the anaphoric strategy. In their globally organized episodes and narrations, some 6- and 8-year olds used WE discourse pragmatic resources, such as subject chaining and left dislocated structures, for emphasis and contrast, but none used these referential structures to background and foreground events to the extent that the older groups did. Three speakers in the youngest group used the least mature strategy, the pronominal strategy, for all or most of their stories. This “here-and-now” picture description mode does not require the discourse planning discussed above (Karmiloff-Smith, 1985; Bamberg, 1987; Wigglesworth, 1997; Severing and Verhoeven, 2001).

The complexity of the prompt effectively drew out differences between the 8- and 6-year-olds and the older groups, as half of the speakers in the younger groups shifted between strategies, particularly in response to the increased cognitive demands of the referencing task (Wigglesworth, 1997; Berman, 2004). Those who had started with a globally organizing strategy abandoned this, and did not produce referring expressions on-line that created local cohesion, or draw on this to create coherence over the stretch or whole text. Rather, they stepped through the story content clause-by-clause, not taking into account the preceding discourse or, apparently, planning ahead to the coming immediate discourse.

The discussion thus far has described the developmental sequence of reference organization in narrative among Wumpurrarni English speaking children and related this to previous findings. It must now be asked what this might tell about the mechanisms for the developmental trajectory. Attention to the planning task in narrations and segments with the nominal strategy as a response to task difficulty, as compared to globally organized segments/narrations, is warranted, as it provides a variable to best bring out differences in performances.

Task difficulty was a factor of story content and complexity for some 6- and 8-year-olds. Their response was to switch to a nominal strategy at points in the narrative, or to simplify story content, or both. The second group who responded to task complexity were some 10- and 12-year olds, as a factor of their code choice. At points, both groups simply depicted the key story content, and like the younger speakers described above, they did not use referring expressions to create local cohesion or global coherence over the stretch or whole text. Speakers who constructed a global stretch or narration, on the other hand, undertook a long-term strategy over the narration as a whole (or attempted to), which was realized at each referential move. Thus, a planning mechanism is manifest in two intersecting ways; as a narrative organizing schema, and in the on-line production of locally cohesive referring expressions. This opens the question of whether two types of cognitive representations are involved: an underlying, abstract, context dependent story schema or a particular representation, which is constructed on-line to communicate a particular story in a particular narrative context. Some have proposed that both are at play and develop at the same time, but do not relate them (Shapiro and Hudson, 1991, 1997). The current study supports the proposal that the two are tightly interdependent, and indeed co-constructing.

The next step is to relate this back to the data. Concentrating on the 6- and 8-year-olds, most started out with a global strategy (thematic), indicating a planning ability at a schema level, with attention to local cohesion. However, faced with task difficulty, three responses were possible: simplify the story content, shift the planning task from a long term plan (a discourse-wide strategy) to the nominal frame-by-frame mode, focus on key story content, or both. The mechanism that emerges then is co-ordination; to plan and manage referential strategy (over greater and shorter stretches), to create story structure and to depict event content. The correlation between a more mature referencing strategy, more detailed event content and story structure, and speaker's age, as a clear developmental finding, is underpinned by this ability to co-ordinate the different tasks in narration. There was a clear difference between the productions by speakers aged eight and six, and the older age groups. Where previous discourse remains a stable unit in a speaker's long-term memory and is accessed easily in the on-line process of generating the semantic representations to be expressed in the current utterance, the story content can be formulated fully and spontaneously, and this is a cognitive and linguistic coordination task. This accords largely with findings by Wong and Johnston (2004) and Mäkinen et al. (2014, p. 37) who write:

when the story structure is mastered and not much processing is needed to maintain coherence, there is more capacity to focus on cohesion. [This lends] support to the hypothesis that the use of accurate referencing and the increase of event content seem to have parallel developmental trends. In other words, the more information there is, the more accurate the reference use becomes.

The examination of the narrations by older speakers in SV invite a reformulation of this: when the linguistic code is mastered and not much processing is needed to maintain coherence, there is more capacity to focus on cohesion (Severing and Verhoeven, 2001; Stavans, 2001).

## Conclusion

To summarize, the study confirmed a developmental trend in the narrative skills of children, through fine-grained investigation of picture prompt generated narrations. The children in the study demonstrated their narrative skill in sophisticated ways in HV, and in ways developmentally akin to children speaking other languages in previous studies. The general developmental trajectory discerned involves an increasing mastery in the cognitive and linguistic task of co-ordination of reference, story structure, and story content. This suggests that development of linguistic forms, along with story content and complexity, are important in investigating development in later childhood.

Conducting research in small speaker populations poses challenges, but their inclusion is crucial to a full account of child language development (Kelly and Nordlinger, 2014). The analysis carried out has drawn on a small data set, and a more comprehensive data set is needed to provide a more thorough account. The small number of SV narrations is clearly a limitation of the present study, and allow observations only. Further investigation of home and school varieties is much needed.

The inclusion of the small set of SV narrations provided evidence that the cognitive load associated with code choice “interrupted” discourse organization for some speakers. This has important implications for bi-varietal speakers in many settings, where the language of education is the student's less dominant code. As educators typically do not have access to children's first language/variety competency, their assessment of children's skill level is based only on their impressions of the children's performance in the school variety, English. The children's ability to construct discourse, partially obscured in second language/variety productions, has been illuminated in this study. It thus contributes to the study of bi-varietal development and learning needs of such speakers locally, where awareness is growing (McIntosh et al., 2012; Sellwood and Angelo, 2012), in particular in the face of high-stakes literacy testing in Australia (Angelo, 2013), and more broadly.

## Funding

This research was supported by Australian Research Council Discovery Grants (DP0343189), as part of the Aboriginal Child Language Acquisition (ACLA) (2003-2007) project.

## Ethics statement

The study was carried out under ethics approval from the Melbourne University Ethics Committee for the ACLA project. Permission to record, analyse and publish results on data was gathered from parents and caregivers of children.

### Conflict of interest statement

The author declares that the research was conducted in the absence of any commercial or financial relationships that could be construed as a potential conflict of interest.

## Footnotes

^1^Glossing abbreviations: Det, determiner; Conj, conjunction; 3S, third person singular; 3Du, third person dual; 3Pl, third person plural; 3SPoss, third person singular possessive; Pl, plural; Pst, past tense form; Trans, transitive marker; Prep, preposition; Dis, discourse marker.

^2^http://languages-linguistics.unimelb.edu.au/past-projects/acla1#aclaoneoverview
